# Correction: Patchouli oil isolated from the leaves of *Pogostemon cablin* ameliorates ethanol-induced acute liver injury in rats *via* inhibition of oxidative stress and lipid accumulation

**DOI:** 10.1039/c8ra90062k

**Published:** 2018-08-14

**Authors:** Qiong-Hui Huang, Xue Wu, Xiao-Hong Chen, Jia-Zhen Wu, Zi-Ren Su, Jia-Li Liang, Yu-Cui Li, Xiao-Ping Lai, Jian-Nan Chen, Yu-Hong Liu

**Affiliations:** Guangdong Provincial Key Laboratory of New Drug Development and Research of Chinese Medicine, Mathematical Engineering Academy of Chinese Medicine, Guangzhou University of Chinese Medicine, Guangzhou Higher Education Mega Center 232# Wai Huan East Road Guangzhou 510006 P. R China yu_hong_liu@126.com chenjiannan@gzucm.edu.cn +86-20-3935-8086; The First Affiliated Hospital of Chinese Medicine, Guangzhou University of Chinese Medicine 12 Airport Road, Bai Yun District Guangzhou 510405 PR China; Dongguan Mathematical Engineering Academy of Chinese Medicine, Guangzhou University of Chinese Medicine Dongguan 523808 PR China

## Abstract

Correction for ‘Patchouli oil isolated from the leaves of *Pogostemon cablin* ameliorates ethanol-induced acute liver injury in rats *via* inhibition of oxidative stress and lipid accumulation’ by Qiong-Hui Huang *et al.*, *RSC Adv.*, 2018, **8**, 24399–24410.

The authors regret that Table 2 was omitted from the original article and that the caption of Fig. 1 in the original article is incorrect. Table 2 and the corrected caption of Fig. 1 are shown below.

**Table 1:** Components of common peaks in fingerprint of patchouli oil

Peak number	Patchouli oil		Components
	Retention time	Relative content (%)	
1	9.101	3.968	β-Patchoulene
2	10.211	15.173	α-Guaiene
3	10.314	7.395	Seychellene
4	10.572	4.505	α-Patchoulene
5	11.296	3.434	Globulol
6	11.396	23.532	Azulene
7	12.649	41.993	Patchouli alcohol

Corrected Fig. 1 caption: GC-MS analysis of patchouli oil. Numbers indicated on peaks correspond to those in Table 2.

The Royal Society of Chemistry apologises for these errors and any consequent inconvenience to authors and readers.

## Supplementary Material

